# Lead Concentrations in Tissues of Pigeons (*Columba livia*) in the Urban Area of Comarca Lagunera, Mexico

**DOI:** 10.3390/toxics12110830

**Published:** 2024-11-19

**Authors:** Andrea Ocampo-Lopez, Cristo Omar Puente-Valenzuela, Homero Sánchez-Galván, Ana Alejandra Valenzuela-García, Josué Raymundo Estrada-Arellano, Ramón Alfredo Delgado-González, Jorge Alejandro Aguirre-Joya, Cristian Torres-León, Alejandra Ocampo-Lopez, David Ramiro Aguillón-Gutiérrez

**Affiliations:** 1 Veterinay Diagnostic Unit, Laguna Unit, Antonio Narro Agrarian Autonomous University, Periférico Raúl López Sánchez, Col. Valle Verde s/n, Torreon 27054, Mexico; ocampoandrea2304@gmail.com (A.O.-L.); raldego@gmail.com (R.A.D.-G.); 2 Faculty of Biology, Juarez University of the State of Durango, Av. Universidad s/n, Fracc. Filadelfia, Gomez Palacio 35010, Mexico; puvacrom@ujed.mx (C.O.P.-V.); hosagafcb@gmail.com (H.S.-G.); ale.valenzuela@ujed.mx (A.A.V.-G.); j.estradarellano@gmail.com (J.R.E.-A.); 3 Research center and Ethnobiological Garden, Autonomous University of Coahuila, Dr. Francisco González 37, Viesca 27480, Mexico; jorge_aguirre@uadec.edu.mx (J.A.A.-J.); ctorresleon@uadec.edu.mx (C.T.-L.); jandisoc@gmail.com (A.O.-L.)

**Keywords:** heavy metals, contamination, pigeon, bioindicator, urban area

## Abstract

The Comarca Lagunera is one of Mexico’s most important productive areas. Its main economic activities are livestock, agriculture, and the processing industry. A wide variety of industries emit wastes that are considered highly toxic environmental pollutants, which have strong negative impacts on public health. The objective of this work was to determine the lead concentrations present in tissues of pigeons (*Columba livia*) belonging to the urban area of the Comarca Lagunera, Mexico. Specimens were collected from the localities that comprise the region and the tissue extracted; the organs were dried, calcined, and diluted in an acidic HCl solution. Lead concentrations were obtained by atomic absorption spectrometry using the graphite furnace technique. The results demonstrate the presence of lead in all the tissues analyzed, with maximum concentrations of 191.14 mg/kg and minimum concentrations of 0.86 mg/kg, the area with the highest average concentration being Torreón, Coahuila (*p* = 0.030). The organ with the highest concentration was the bone (*p* = 0.000). Evidence of lead poisoning is presented in *Columba livia* tissues in the Comarca Lagunera, thus demonstrating the presence of this contaminant and the ability of these pigeons to function as bioindicators of environmental contamination.

## 1. Introduction

The environment comprises the sum of biotic factors, which describe living organisms, from plants and animals to microorganisms, and abiotic factors, comprising those non-living entities vital for the development of biotic factors [1]. These factors are in states of equilibrium and exchange on a continuous basis, but can be altered by the introduction of harmful substances to living entities, leading to contamination of the environment and increased risk of disease [2]. Pollution is defined as the introduction of any harmful substance, whether in solid, liquid, or gaseous form, which at a certain concentration alters the environment into which it is introduced [1]. At present, environmental pollution is one of the problems with the greatest impact on the environment, not only because of the alterations caused in the environment but also because of the effect it has on public and individual health; contact with this type of pollutant can represent an important risk factor for the increase in morbidity and mortality in different populations of living beings [3]. Large cities and metropolitan areas face serious pollution problems due to overcrowded places, high flows of motorized vehicles, and unplanned industrial developments [4]. The effects of exposure to pollutants affect not only current but also future generations, bringing with them health problems such as cancers, reduced fertility, respiratory problems, and various inflammatory disorders, among others [5,6]. There are different types of pollution. In the case of air pollution, the primary sources come from industrial activities, burning fossil fuels, and various wastes [7]. Large industrial complexes constitute stationary sources of various pollutants such as fine dusts, sulfur and nitrogen dioxide, carbon monoxide, ozone, volatile compounds, and heavy metals [8].

The Comarca Lagunera, southwest of Coahuila and northeast of Durango, is one of Mexico’s most important productive areas. Its main economic activities are livestock, agriculture, and the processing industry. Anthropogenic activities in the region have led to increased environmental pollution, bringing with them a growing need for monitoring, remediation, and prevention measures [9]. The Comarca Lagunera is home to the world’s largest silver refining plant, the leading producer of gold and lead in Latin America. The metallurgical complex in the region is composed of lead smelting and lead–silver refining and has a capacity of 118,000 tonnes of bullion in the lead smelter [10].

Heavy metals have been present on Earth since its formation, but their concentrations have increased in terrestrial and aquatic environments in an emergent manner due to anthropogenic activities. They are defined as metals and metalloids with densities above 5 g/cm^3^, with bioaccumulate potential once in food chains, and commonly with high toxicity to living organisms that come into contact with them [11]. Media such as soil and air and accumulation in living organisms can reflect heavy metal exposure to the population, with inhalation, ingestion, and direct skin contact being the usual routes of entry [12]. Heavy metals have a natural origin on our planet, but their concentrations have started to fluctuate and increase due to human activity. Many have harmful effects on the health of living beings, which have access to them through inhalation, ingestion, and contact. Once inside the organism, they cause various types of physiological damage. Among the most important metals with negative health impacts are lead, cadmium, arsenic, chromium, aluminum, iron, and mercury [13]. Lead is a toxic element that can accumulate in blood and bone and reach significant concentrations in the kidneys, liver, brain, and skin; exposure to it can cause nervous system, circulatory, gastrointestinal, and hormonal problems [14]. Among the most common anthropogenic activities through which lead can be found in the environment are mining, waste from the battery industry, and its use as an additive to substances such as gasoline, paints, and varnishes, as well as the raw material processing industry, including mining resources [15]. In nature, lead does not exist in pure form; it is part of the components of other minerals, the predominant example being galena, where silver is mostly found [16].

The main routes of entry of lead into the body are considered to be digestive, respiratory, and cutaneous [17], and it also has an important impact at the gestational level due to its ability to cross the placental barrier in mammals and its ability to mobilize during the laying season in birds together with the calcium that makes up the eggshell [18]. It plays an important toxic role by substituting for calcium in the processes in which it is involved, thus affecting normal neuronal functions such as the release of neurotransmitters, causing excitotoxicity and alteration of myelin synthesis [19]. This calcium substitution process also causes alterations in bone composition; it can alter bone homeostasis by competing with calcium for its place in apatite networks, and once this metal is absorbed, it is stored in mineralized tissues [20]. In the case of the liver, it is considered one of the major soft organ accumulators of lead and can have an acute and chronic presentation [21]; when exposure to this substance is chronic, it causes dyslipidemia, hormonal depression, and hypercholesterolemia [22].

The pigeon *C. livia*, also called rock pigeon, is a bird species with an average size ranging from 30.5 to 35.5 cm wing length and a weight of 180–355 g; it does not present sexual dimorphism, and its plumage varies between a light grey pattern and two large blackish stripes on the wings, white rump, and purple and green iridescence on the neck; it is possible to find some species where these color characteristics are not met. The shades can range from white to brown [23]. Pigeons of the species *Columba livia* have a high potential as environmental bioindicators; it is a species that is distributed worldwide and is even considered urban fauna, although it is possible to find them in feral and domesticated states, given that their populations are abundant in cities and rural areas. They are considered suitable bioindicators for measuring environmental health because they do not migrate easily from their nesting sites. From the conditions in which the concentration of Pb in these individuals is found, the presence of this and other pollutants in different animal species can be assumed, functioning as a reflection of the environmental conditions of the urban area of the Comarca Lagunera, Mexico, and as a method of monitoring possible risks to human health and the affectation of the diversity of local ecosystems.

Several studies, such as the one conducted by Delgado [24] in Mexico City, demonstrate the ability of *C. livia* to resist these pollutants and reflect the environmental condition. Nam [25] used domestic pigeons of the species *C. livia* from urban and industrial areas to measure the concentration of lead and cadmium in the city of Seoul, South Korea, using the bone, kidney, liver, and lung for analysis, reporting lead concentrations of 29.5 ± 21.1 μg/wet weight, 4.13 ± 1.31 μg/wet weight, 2.33 ± 0.78 μg/wet weight, and 1.72 ± 0.66 μg/wet weight, respectively, in adults. In 2010, in the city of Rabat-Salé, Morocco, a study was carried out to measure air pollution by heavy metals using feral pigeons as bioindicators, determining the concentration of these metals through samples of kidney, liver, lung, heart, and blood tissue, reporting the highest concentration of lead and cadmium in kidneys (0.56 ± 0.06 μg/kg and 3.07 ± 0.78 μg/kg, respectively) [26]. Begum and Sehrin [27] evaluated heavy metal levels in birds of this species and focused on human consumption, where concentrations of 0.067 ± 0.52 μg/g of lead were obtained. In 2021, Valladares [28] carried out an analysis of lead, cadmium, and arsenic content in the liver and bone of *C. livia* pigeons found in areas known to be previously contaminated with mining waste, reporting averages of 17.027 μg/g and 108.436 μg/g of lead, respectively.

Considering this background, the Comarca Lagunera being a region with high environmental pollution, and that there are no studies in this area of bioindicators of heavy metal levels using birds, the purpose of the present research is to determine the levels of lead in tissues of domestic pigeons.

## 2. Materials and Methods

### 2.1. Study Site

**Figure 1:**
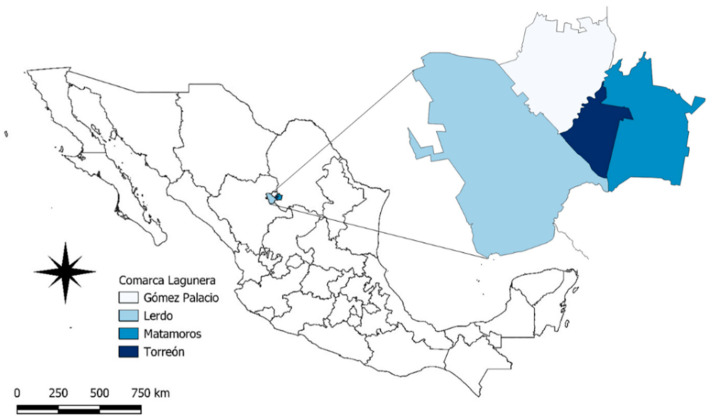
Sampling points in the Comarca Lagunera, Mexico.

The Comarca Lagunera is located between the states of Durango and Coahuila, has an extension of more than 44,887 km^2^, and most of it is in the Chihuahuan Desert [29]. It comprises 15 municipalities, 4 of which were selected for trapping (Figure 1). It is one of the ten most productive areas in the country and has more than 1.2 million inhabitants [30]. Its temperatures are warm, with characteristic semi-dry temperate climates in the higher elevations and dry temperate climates in the lower elevations; in the middle elevations, the climate is very dry and semi-warm [31], which corresponds to the classification BWhw” (e’) [32].

### 2.2. Biological Model

The pigeon *C. livia* is an invasive bird native to Africa and Eurasia, belonging to the Columbidae family (Table 1), with an excellent adaptive system to human environments that allows it to have very high population growth [33]. Its close relationship with humans stems from the anthropogenic food source and nesting sites provided by urban constructions [34]. The pigeons have demonstrated a wide potential as bioindicators and have been one of the most widely used biological models in determining metals in tissues [35].

### 2.3. Fieldwork

Birds were taken from each of the municipalities (Gómez Palacio, Durango, *n* = 25; Lerdo, Durango, *n* = 12; Torreón, Coahuila, *n* = 16; Matamoros, Coahuila, *n* = 12); a specific area was chosen for sampling based on the criteria of the number of people and businesses in the area, its central location in each city sampled, and the fact that they are wooded areas and have the necessary urban infrastructure for the presence of *C. livia* pigeon populations. These points are public squares with similar climates and flora. In order to carry out the capture activities, a permit was obtained from the Ministry of the Environment and Natural Resources (SEMARNAT), obtaining the folio SPARN/DGVS/06267/22. The captures were carried out during daylight hours from 20 July to 12 August 2022. The collection sites and their coordinates are shown below:

- Parque Victoria, Lerdo, Durango (25°32′33″ N 103°31′23″ W).
- Plaza de Armas, Gómez Palacio, Durango (25°34′05″ N 103°30′00″ W).
- Alameda Zaragoza, Torreón, Coahuila (25°32′21″ N 103°26′43″ W).
- Plaza Mayor, Matamoros, Coahuila (25°31′41″ N 103°13′46″ W).

The specimens were captured using manual nets and carried out by a collection brigade of two people from 20 to 30 June 2022, from 9:00 to 13:00 h. The specimens were taken the same day of their capture to the Pathology Laboratory of the Diagnostic Unit of the Universidad Autónoma Agraria Antonio Narro (Torreón, Mexico), Laguna Unit, where they were processed.

### 2.4. Specimen Processing

Birds belonging to all groups collected were slaughtered by cervical dislocation in accordance with the provisions of the Mexican Official Standard for the Humane Slaughter of Domestic and Wild Animals (NOM-033-ZOO-2014). The primary necropsy procedure was performed using a dissection kit. Brain, lung, liver, kidney, and bone were extracted using plastic tools; organs that were not processed during this study were preserved in 5% formalin and frozen (0 to 4 °C) for further studies. During necropsy, identification of the reproductive organs of the specimens was carried out to determine sex.

### 2.5. Sample Processing and Analysis

**Figure 2:**
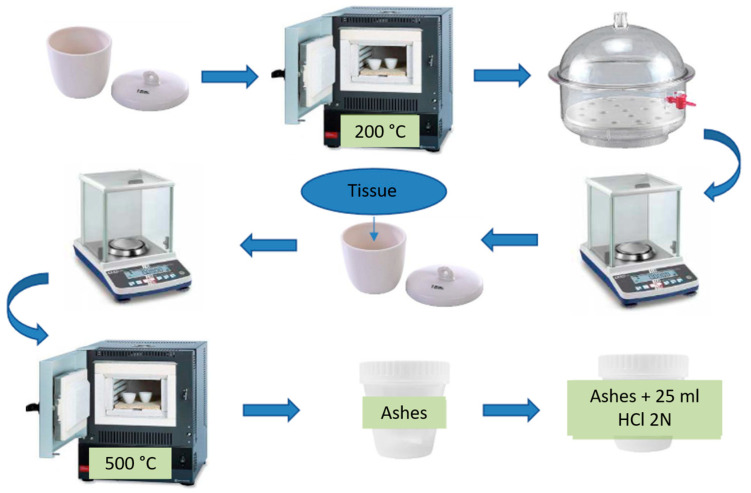
Sample processing and analysis.

Porcelain crucibles were brought to constant weight at a temperature of 200 °C for 2 h in an oven, after which they were left to stand in a desiccator for 2 h, and their weight was recorded. The organs chosen for the study were dried using an oven at 105 °C and handled in the crucibles at constant weight. The weight of each organ was recorded, and they were then incinerated at 500 °C for 12 h in the muffle. After this period, the resulting ashes were suspended in 25 mL of HCl solution with a 2N concentration following the methodology of Delgado [24]. The ashes suspended in 2N HCl were diluted using distilled water to concentrations favoring their reading in the spectra (Figure 2). Analysis of the concentrations was performed using graphite furnace atomic absorption spectrophotometry, the blanks were Tritón 100x and array modifier, and the 2N HCl was also used as a blank during the analysis to check the presence of contamination of samples. This study used a NIST 1577b bovine liver sample, with a recovery of >92%, a quantification detection limit of 10 ng/g, and a detection limit of 3 ng/g.

### 2.6. Statistical Analysis

Statistical analysis was performed using Kruskal–Wallis and post hoc Mann–Whitney. Normality tests were performed using Shapiro–Wilk and Kolmogorov–Smirnov. The statistical program used was GraphP 10.1.1.

## 3. Results

**Figure 3:**
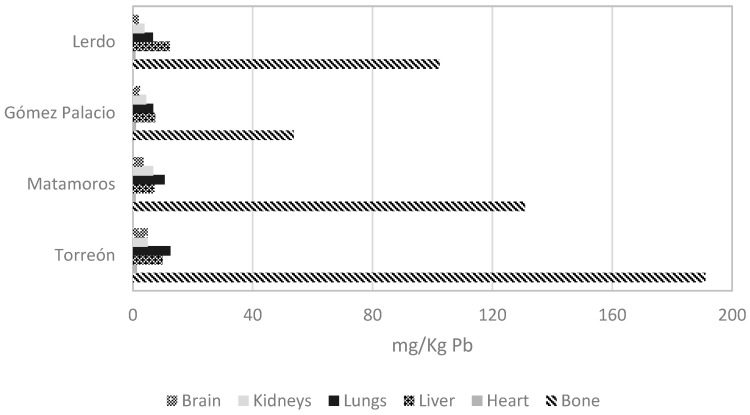
Comparison of lead concentration among all organs and cities. The concentration in bones is more elevated compared with the soft tissues.

**Figure 4:**
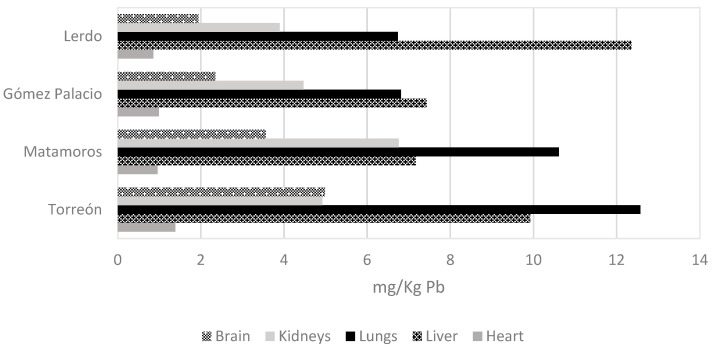
Comparison of lead concentration among soft tissues.

**Figure 5:**
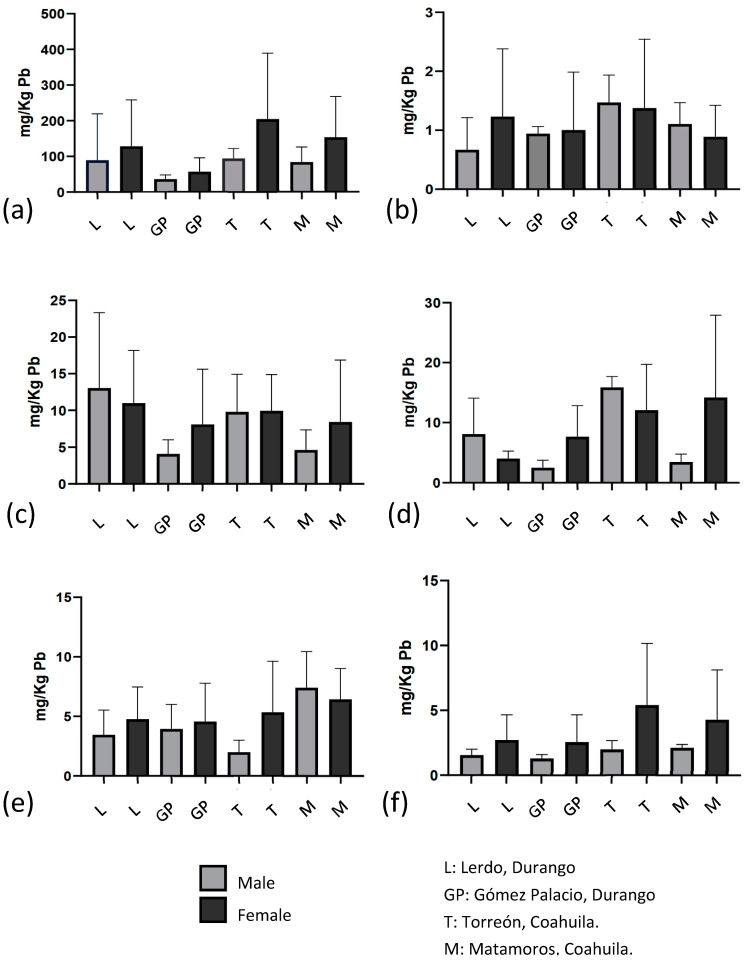
Lead concentrations in tissues of *C. livia*. (**a**) Bone, (**b**) Heart, (**c**) Liver, (**d**) Lung, (**e**) Kidney, (**f**) Brain.

Sex organ analysis determined that of the 64 pigeons collected, 16 (25%) were male, and 48 (75%) were female. The average weight of the birds was 264.5 g, with a minimum of 185 g and a maximum of 340 g. Figure 3 compares all the samples’ concentrations, with bone being the organ with the highest concentrations of all and liver and lung when comparing soft organs; Figure 4 compared only soft tissues. Torreón, Coahuila, is the city with the highest values in bone, heart, lungs, and brain; Lerdo, Durango, is the city with the highest average lead concentration in liver; the average lead concentration in kidney was highest in the city of Matamoros, Coahuila, compared to the other cities. There is lead concentration in all tissues and cities sampled. The concentrations of metals are shown in Figure 5 by organ, city, and bird sex. The tissue and city with the highest lead concentration were bones (*p* = 0.000) and Torreón Coahuila (*p* = 0.030), respectively, using the Kruskal–Wallis test and a Mann–Whitney post hoc.

## 4. Discussion

The presence of lead was found in the different tissues of *Columba livia* specimens collected in the region, which indicates that other species of urban fauna could probably be affected by heavy metals, and that *Columba livia* pigeons function as bioindicators of environmental pollution. This work coincides with that reported by Delgado [24] on the use of the *Columba* livia pigeon as a bioindicator in our country, being the first and only record in Mexico, and is very close to the results obtained by Valladares [28] in Chile and Guevara-Torres [35] in Peru. One of the previous works carried out in the region with other biological models is that of Domínguez-Zuñiga [37], who analyzed lead concentrations in fungi with urban growth in the region, finding that Torreon, Coahuila, was the city most affected by this pollutant, where lead concentrations were found in various species of fungi collected in different parts of the city.

The organ with the highest lead concentration was bone, where the maximum value was 191.04 mg/kg; these results are similar to those found by Valladares [28], where *Columba livia* pigeons were used to measure the concentrations of lead and other metals in an area contaminated by mining in the city of Arica, Chile; the two areas sampled share a history of contamination by mining or related activities, and it is an urban area inhabited not only by numerous people but also by a great diversity of urban species such as this pigeon. Physiologically, when lead exposures occur over long periods, there is a cumulative trend in the bones of the bird throughout its life [38]; this could be an indication of chronic exposure to this pollutant for individuals living in the sampled areas. Some authors explain this type of accumulation in bone in different animal models as the product of exposure in recurrent periods; however, in nature, the *Columba livia* pigeon does not achieve such a long life. According to Ishii [39], lead concentrations in the trabecular bones of birds increase more rapidly than in other bones due to the activities of lead in blood. Trabecular bones are tissues highly irrigated from blood vessels, and have an interaction within the exchange of calcium, iron, and magnesium, minerals that lead has the ability to substitute for in the bones for their chemical characteristics like divalent cation charge [40]. This could suggest this bone is the tissue with the greatest potential for lead accumulation in the face of prolonged exposure, which is consistent with what has been reported by Lee [41], where the bone and eggshell where the tissues with the higher concentration of lead.

The average concentrations of lead in the heart were higher than those reported by Elabidi [26] in Rabat-Sale, Morocco, suggesting that this contaminant is present in this way because this organ cannot filter substances. At present, there are not many studies that focus on the analysis of heart tissue of the pigeon *Columba livia*, but there are studies on other bird species, such as the one carried out by Millaku [42], where they analyzed hearts of house sparrows (*Passer domesticus*) and obtained concentrations higher than those found in this work. This study shares with ours the historical characteristics of contamination of the areas sampled by mining activities involving lead. However, it uses another species of bird that does not have an ethology like that of *C. livia* pigeons, which, unlike *Passer domesticus*, do not travel long distances, and their behavior is more gregarious, which may be the reason why the two biological models, despite being birds, present variations in their ability to accumulate metals in this tissue.

The average maximum lead concentration in the liver was 12.36 mg/kg; these results are higher than those found by Guevara-Torres [35], who analyzed specimens of *Columba livia* from the city of Lima, Peru, and reported an average of 0.624 mg/kg in the analysis of this tissue. This difference could be explained due to the disparity in the sources of contamination considered in the two studies. In their work, Guevara-Torres [35] analyzed a city polluted by vehicular influx and a variety of industries, unlike our study and others that compared that focus on the analysis of areas polluted by mining or related activities, and this is also the possible reason why higher results are presented than the analysis done by Romero [43], where specimens were affected systemically by lead bullets. In contrast to this, Valladares [28], who worked with *Columba livia* pigeons from an area contaminated by mining (City of Arica, Chile) and with whom the characteristics of the sources of these pollutants are shared, found similar results with average lead concentrations of 13.007 and 21.084 mg/kg.

For the lungs case, the lead concentrations that were found are higher than those reported by Delgado [24] in their study, carried out in Mexico City. The average lead concentrations were 3.65 mg/kg, while the average values obtained in our work were 12.57 mg/kg, which could suggest the inhalation route as the main entry access of this pollutant in the *Columba livia* pigeons inhabiting the region.

Regarding the average concentrations of lead in the kidney, Lee [41] reported in their study that the kidney is the second organ where lead accumulates the most (the first being bone), with concentrations of 2.116 mg/kg, a presentation three times lower than that obtained in our work with averages of 6.76 mg/kg. These studies are similar in the choice of rural areas as sampling points; the difference between the concentrations could be due to the disparity in the sources of contamination. Delgado [24] found similar results to those in this work, with maximum average concentrations of 7.61 mg/kg in *Columba livia* pigeons sampled in Mexico City, Mexico.

The average concentration of lead in the brain of *Columba livia* was 4.98 mg/kg; this result is lower than that obtained by Delgado [24], where the average concentration of lead in this tissue in Mexico City was 7.5 mg/kg. Both works share the development methodology, but there are differences in the sources of contamination sampled. Mexico City has a much higher population influx than the Comarca Lagunera, even in those years, which could be the reason for this difference. At present, there are not many studies that analyze lead concentrations in the brains of *Columba livia*. Aloupi [44] analyzed the livers and brains of nine different species of waterfowl from the Evros Delta National Park in Greece, but none reached the levels found in this study.

Among the four cities analyzed within the Comarca Lagunera, it was found that there was a significant difference in the city of Torreón, Coahuila, in relation to the rest of the cities; no significant statistical difference was found among the sampled localities. However, the maximum values of this pollutant were found in three of the six organs belonging to the individuals sampled in this city. Torreón, Coahuila, has one of the most important sources of lead emissions in northern Mexico (mining and metallurgical industry). This is the possible reason why in this and other studies, it is considered to be the city with the highest concentrations of this pollutant in the Comarca Lagunera; also, in this region, there is an import background of lead toxicosis in children, where the reported concentration have decreased over time [45].

In the case of Lerdo, Durango, in our work, it was reported as the second city with the highest lead concentrations in our model. However, Domínguez-Zuñiga [37] reported it as the third; Gómez Palacio, Durango, was, for them, the second city with the highest impact of lead contamination. This disparity can be explained by the difference in the chosen biological models and by a geophysical issue of the region. The possible existence of an unknown secondary source of contamination near the city of Lerdo, Durango, which could affect lead levels in these organisms, is considered.

The city of Matamoros, Coahuila, showed lower lead concentrations than Torreón, Coahuila, and Lerdo, Durango, which had the highest average values for lead concentration in kidneys. Currently, there is no previous record of analysis of lead concentrations in the city of Matamoros, Coahuila, using biological models.

In Gómez Palacio, Durango, lead concentrations were detected in all tissues of all pigeons analyzed. Domínguez-Zuñiga [37] found concentrations of 100.49 mg/kg in mushrooms belonging to the species *Coprinellus truncorumn* found in this municipality. Santoyo-Martinez [46] reported maximum concentrations of lead in dusts sampled outdoors of 464 mg/kg, which could suggest the presence of this pollutant in the city’s environment regularly. The organ with the highest average lead concentration in this municipality was bone, with a value of 53.74 mg/kg, although this concentration does not reach the bones of other *Columba livia* pigeons analyzed in this study. Likely, exposure to lead in this area is not as chronic as in the rest of the cities but recurrent.

## 5. Conclusions

Lead was found in all *C. livia* tissues analyzed and at all sampling sites. The organ with the highest lead concentration was bone; a significant difference was found in this tissue compared to the others. The city with the highest lead concentration in individuals was Torreón, Coahuila, which is significantly different from other cities. In the case of the municipality of Matamoros, these results represent the first report of lead contamination in animal models. It also proves the ability of *C. livia* to function as bioindicators of environmental contamination.

## Figures and Tables

**Table 1:** Taxonomic classification of *Columba livia* [36].

Kingdom	Animalia
Filum	Chordata
Class	Birds
Order	Columbiformes
Family	Columbidae
Subfamily	Columbinae
Genus	*Columba*
Specie	*Columba livia*

## Data Availability

The raw data supporting the conclusions of this article will be made available by the authors on request.
